# The effect of sertraline on emotional processing: secondary analyses of the PANDA randomised controlled trial

**DOI:** 10.1017/S0033291720004985

**Published:** 2022-10

**Authors:** Norin Ahmed, Jessica K. Bone, Gemma Lewis, Nick Freemantle, Catherine J. Harmer, Larisa Duffy, Glyn Lewis

**Affiliations:** 1 Division of Psychiatry, University College London, Faculty of Brain Sciences, London, UK; 2 Institute of Clinical Trials and Methodology, University College London, London, UK; 3 University Department of Psychiatry, Warneford Hospital, Oxford, UK; 4 Oxford Health NHS Foundation Trust, Warneford Hospital, Oxford, UK

**Keywords:** Antidepressants, cognitive neuropsychological model, depression, emotional processing, memory, recall

## Abstract

**Background:**

According to the cognitive neuropsychological model, antidepressants reduce symptoms of depression and anxiety by increasing positive relative to negative information processing. Most studies of whether antidepressants alter emotional processing use small samples of healthy individuals, which lead to low statistical power and selection bias and are difficult to generalise to clinical practice. We tested whether the selective serotonin reuptake inhibitor (SSRI) sertraline altered recall of positive and negative information in a large randomised controlled trial (RCT) of patients with depressive symptoms recruited from primary care.

**Methods:**

The PANDA trial was a pragmatic multicentre double-blind RCT comparing sertraline with placebo. Memory for personality descriptors was tested at baseline and 2 and 6 weeks after randomisation using a computerised emotional categorisation task followed by a free recall. We measured the number of positive and negative words correctly recalled (hits). Poisson mixed models were used to analyse longitudinal associations between treatment allocation and hits.

**Results:**

A total of 576 participants (88% of those randomised) completed the recall task at 2 and 6 weeks. We found no evidence that positive or negative hits differed according to treatment allocation at 2 or 6 weeks (adjusted positive hits ratio = 0.97, 95% CI 0.90–1.05, *p* = 0.52; adjusted negative hits ratio = 0.99, 95% CI 0.90–1.08, *p* = 0.76).

**Conclusions:**

In the largest individual placebo-controlled trial of an antidepressant not funded by the pharmaceutical industry, we found no evidence that sertraline altered positive or negative recall early in treatment. These findings challenge some assumptions of the cognitive neuropsychological model of antidepressant action.

## Introduction

Common mental health problems including depression and generalised anxiety disorder (GAD) are leading causes of disease burden worldwide (World Health Organization, 2018). People with depression are usually treated in primary care and selective serotonin reuptake inhibitor (SSRI) antidepressants are often the first-line treatment (Lewis et al., 2019). Antidepressant use has risen dramatically in high-income countries over the past two decades. In the USA in 2011–2014, 12.7% of people aged 12 and over reported using antidepressants in the past month, compared to 7.7% in 1999–2002 (Pratt, Brody, & Gu, 2017).

Despite the widespread use of SSRIs, little is known about their mechanisms of action, beyond the initial effect on serotonin. The cognitive neuropsychological model proposes that antidepressants improve symptoms of depression and anxiety by altering emotional processing (Harmer, Duman, & Cowen, 2017; Reinecke & Harmer, 2016; Roiser, Elliott, & Sahakian, 2012). Emotional processing refers to how people perceive, interpret, and remember their environment. Depression and anxiety have been associated with reduced positive, or increased negative, emotional processing (Hayes & Hirsch, 2007; Roiser & Sahakian, 2017). Antidepressants may increase the processing of positive relative to negative information early on in treatment, within hours or days, before any change in symptoms (Harmer et al., 2017; Reinecke & Harmer, 2016; Roiser et al., 2012). This is hypothesised to be a common pathway across different classes of antidepressants, regardless of their biological mechanism of action.

Memory is an important aspect of emotional processing. Self-referential recall can be tested by asking individuals to classify personality characteristics as positive or negative, followed by a surprise recall test, in which participants are asked to remember as many characteristics as possible. There is longitudinal evidence of an association between reduced positive self-referential recall and increased depressive symptoms (Lewis et al., 2017).

Self-referential recall tasks have also been used to compare the effects of antidepressants to placebo in randomised designs. Most studies have administered one dose of an antidepressant compared with placebo to healthy individuals and measured recall within a few hours. However, the evidence from these studies is inconsistent. Some report no evidence of an effect of antidepressant compared to placebo on recall (Browning, Reid, Cowen, Goodwin, & Harmer, 2007; Harmer et al., 2013; Komulainen et al., 2016; Walsh et al., 2018). Others report increased positive, reduced negative, or an improved ratio of positive to negative recall in people taking antidepressants (Arnone, Horder, Cowen, & Harmer, 2009; Cooper, Whiting, Cowen, & Harmer, 2015; Gibbs, Bautista, Mowlem, Naudts, & Duka, 2013, 2014; Harmer et al., 2003, 2008). In studies of healthy individuals, those taking citalopram, reboxetine, or 25 mg (but not 50 mg) agomelatine for a longer period (7–8 days) showed increased positive versus negative recall compared to placebo (Harmer, Shelley, Cowen, & Goodwin, 2004; Harmer et al., 2011).

Few studies have tested the effect of antidepressants on recall among people with depressive or anxiety symptoms. In one study, a single dose of reboxetine (compared with placebo) led to increased recall of positive words in people with depression, but there was no evidence for an effect in healthy individuals (Harmer, O'Sullivan, & Favaron, 2009). One other study of people with depression administered escitalopram or placebo for 7 days but found no evidence of a difference in recall (Komulainen et al., 2018).

Although previous studies of recall have not consistently supported the cognitive neuropsychological model, these RCTs have been small, with a maximum of 34 participants per study arm (Gibbs et al., 2013). This may limit statistical power and affect validity (Button et al., 2013). As most studies include either healthy individuals or people diagnosed with depression, their findings do not generalise to primary care, where most antidepressants are prescribed. Individuals with mild to moderate symptoms that do not meet diagnostic criteria are often prescribed antidepressants (Kendrick et al., 2009). It is therefore important to investigate the whole range of depression and anxiety symptoms (Bjelland et al., 2009), particularly as there is evidence that antidepressants are effective across the entire range of symptom severity (Lewis et al., 2019).

We analysed data from a large pragmatic double-blind RCT comparing the antidepressant sertraline with placebo in patients with depressive symptoms of any severity or duration, recruited from primary care (Lewis et al., 2019). There was strong evidence that sertraline led to reduced anxiety symptoms and self-reported improvements in mental health within 6 weeks. Evidence of an effect on depressive symptoms took longer to emerge and was more modest, only becoming apparent by 12 weeks (Lewis et al., 2019). We tested whether treatment allocation was associated with positive and negative recall 2 and 6 weeks after randomisation. In line with the cognitive neuropsychological model, we expected sertraline to increase recall of positive information and decrease recall of negative information.

## Methods

### Study design and participants

The PANDA trial (“what are the indications for Prescribing ANtiDepressAnts that will lead to a clinical benefit?”) was a pragmatic multicentre double-blind placebo-controlled randomised trial (Lewis et al., 2019; Salaminios et al., 2017). The original aim of the trial was to test the clinical effectiveness of sertraline in primary care and the role of depression severity and duration. The trial was registered with EudraCT, 2013-003440-22 (protocol number 13/0413; version 6.1) and ISRCTN (reference ISRCTN84544741).

Patients aged 18–74 years were recruited from 179 primary care practices in four UK sites (Bristol, Liverpool, London, York). Patients were eligible if there was uncertainty from both general practitioner (GP) and patient about the possible benefit of an antidepressant. We did not restrict eligibility by specifying lower or higher thresholds of depression severity but relied instead on clinical uncertainty, aiming to improve generalisability to the population receiving antidepressants in primary care. Exclusion criteria were: unable to understand or complete study questionnaires in English; antidepressant treatment in past 8 weeks; comorbid psychosis, schizophrenia, mania, hypomania, bipolar disorder, dementia, eating disorder, or major alcohol or substance abuse; and medical contraindications for sertraline. Further details of the methods and results have previously been published (Lewis et al., 2019; Salaminios et al., 2017).

### Measures

#### Incidental recall task

Words were initially presented in a computerised emotional categorisation task (ECAT). Twenty likeable (e.g. cheerful) and 20 dislikeable (e.g. hostile) personality characteristics were each seen on a computer screen for 500 ms. These positive and negative words were matched according to length, ratings of usage frequency, and meaningfulness. Words were seen in a random order and, after each word, participants chose whether they would ‘like’ or ‘dislike’ to hear someone describing them in this way. Immediately afterwards, participants recalled as many words as possible in 2 mins, speaking them aloud to a researcher.

This task was completed at baseline and 2 and 6 weeks after randomisation. It was a surprise test at baseline, but participants would have known to expect the recall element at follow-ups. We did not expect this to be an issue as this procedure was also used in the PANDA cohort study and did not lead to a marked improvement in recall (Lewis et al., 2017). Each time participants completed the task a different set of 40 personality descriptors were presented. Counts of positive and negative words accurately recalled (hits) and falsely recalled (false alarms) were recorded.

#### Other measures

Demographic variables including age, sex, ethnicity, and marital status were reported at baseline. We used a five-item self-report adherence scale and a binary variable indicating at least 80% adherence (Lewis et al., 2019).

The Clinical Interview Schedule-Revised (CIS-R; Lewis, Pelosi, Araya, & Dunn, 1992) was completed at baseline and is a self-administered, computerised, structured interview measuring 14 common mental disorder symptom groups. The CIS-R was used to generate total symptom severity, depression, and GAD diagnoses meeting International Classification of Diseases, Tenth Revision (ICD-10) criteria, depression duration, and depressive symptom severity. Participants also reported previous depression and antidepressant prescriptions.

The Generalised Anxiety Disorder Assessment (GAD-7; Spitzer, Kroenke, Williams, & Löwe, 2006) was completed at all times and is a 7-item self-report measure of generalised anxiety symptom severity over the past 2 weeks.

### Randomisation and blinding

Participants were individually randomised to sertraline or placebo by PRIMENT Clinical Trials Unit using a remote computer-generated code, stratified by symptom severity, duration of depression, and study site, with random block length. Pre-specified thresholds for stratification were CIS-R total score at baseline (0–11, 12–19, ⩾20) and depression duration at baseline (<2 years or ⩾2 years). Sertraline and placebo were encapsulated and identical in appearance. Participants and researchers were blind to treatment allocation but analyses for this study were not blinded.

### Procedures

Participants were asked to take one capsule a day (50 mg sertraline or placebo) for 1 week and then increase to two capsules a day (100 mg sertraline or placebo). If patients had not responded to treatment after 6 weeks, an increase to three capsules per day could be approved by the principal investigator. Baseline assessments were completed before randomisation and follow-ups took place 2, 6, and 12 weeks after randomisation. Data from baseline, 2 and 6 weeks were used in this study.

### Ethical approval

Ethical approval was obtained from the National Research Ethics Service Committee, East of England – Cambridge South (ref: 13/EE/0418). All participants provided written informed consent. All procedures complied with the ethical standards of the relevant committees on human experimentation, the Helsinki Declaration (2008 revision), and the General Data Protection Regulation. The results presented here focus on the effect of sertraline on recall and are part of a larger program (NIHR RP-PG-0610-10048).

### Statistical analysis

Baseline variables were explored according to treatment allocation. We first checked that participants understood the words presented in the ECAT by analysing the percent of ‘correct’ responses at baseline (online Supplement). We then assessed performance in the recall element of the task. Consistent with previous studies using this task, we analysed positive and negative words which were correctly recalled (e.g. Harmer et al., 2013; Lewis et al., 2017). Analyses using signal detection theory were not considered appropriate because the recall task did not include a binary decision between a target word and a distractor. Instead, participants were asked to list as many words as they could remember from the ECAT.

First, we tested the influence of treatment allocation on positive or negative hits 2 and 6 weeks after randomisation. We used Poisson mixed models with separate models for positive and negative hit outcomes. Time-point (2 or 6 weeks) was clustered within individual and we included random intercept terms for individuals. Next, we reshaped the data so that we could model total hits (positive and negative combined) as a single outcome and word valence (positive versus negative) as an exposure. This allowed us to test statistically for a difference in the influence of sertraline on positive versus negative words, by calculating an interaction between word valence and treatment allocation. These data were also modelled with a Poisson mixed model with random intercepts for individuals.

In order to analyse positive and negative hits on this recall task, analysis of variance (ordinary least squares, under Gaussian assumptions) would often be used, testing whether recall differed according to word valence. However, given that hits were count variables which followed a Poisson distribution, Poisson regression was considered more appropriate. The Poisson models, which have a log(e) link function, allowed us to calculate a hits ratio through exponentiating the parameter estimate. The hits ratio can be interpreted as the ratio of hits in the sertraline group relative to the placebo group. A hits ratio larger than one meant that hits were higher in the sertraline group. Using Poisson mixed models allowed us to include repeated measures within individuals, as hits were measured at 2 and 6 weeks, increasing statistical power and precision of our estimates (Diggle, 1988). Models are presented before and after adjustment for randomisation stratification variables (CIS-R total score, duration of depression, site), time, baseline positive and negative hits, positive and negative false alarms at all times, and variables which were imbalanced at baseline (sex and marital status). Adjusting for false alarms accounted for participants' general tendency to say words which had not previously been presented.

In sensitivity analyses, we tested whether the effect of sertraline on recall differed according to time by including an interaction between treatment allocation and time in all fully adjusted models. We also tested whether the effect of sertraline on recall differed according to baseline depression or anxiety symptoms by including an interaction between treatment allocation, valence, and baseline CIS-R depressive symptom score or baseline GAD-7 score in the fully adjusted model. Other sensitivity analyses are reported in the online Supplement. All analyses were performed using Stata 16 (StataCorp, 2019).

## Results

A total of 655 participants were randomised, 326 (50%) to sertraline and 329 (50%) to placebo. Of these, 79 participants (42 from the sertraline group and 37 from placebo) did not complete the recall task at 2 and/or 6 weeks. This left 576 participants (284 sertraline and 292 placebo) for analyses. Completion of the recall task did not differ statistically by treatment allocation (*p* = 0.73). Missing data were more common in participants who were recruited from London, younger, ethnic minority, single, having financial difficulties, and those who had no formal qualifications, higher baseline depressive symptoms, and higher GAD-7 and life-event scores (Lewis et al., 2019).

Characteristics of the sample overall and according to study arm are shown in Table 1. Participants' mean age was 40.02 years (s.d. = 14.77) and 333 (58%) were female. On the CIS-R, 306 (53%) met ICD-10 criteria for depression and 255 (44%) met GAD criteria. CIS-R depressive symptom scores ranged from 0 to 20, mean 10.43 (s.d. = 4.81). GAD-7 scores ranged from 0 to 21, mean 9.16 (s.d. = 5.20). In total, 346 (60%) patients reported previous antidepressant use. Baseline characteristics of the sample were similar across study arms (Table 1).

**Table 1.** Baseline characteristics for the sample who completed the emotional processing task at 2 or 6 weeks, according to treatment allocation

	Overall (*N* = 576) *n* (%)	Placebo (*n* = 292) *n* (%)	Sertraline (*n* = 284) *n* (%)
Site			
Bristol	237 (41)	120 (41)	117 (41)
Liverpool	107 (19)	54 (19)	53 (19)
London	110 (19)	59 (20)	51 (18)
York	122 (21)	59 (20)	63 (22)
Age			
18–34	228 (40)	117 (40)	111 (39)
35–54	235 (41)	122 (42)	113 (40)
55–74	113 (20)	53 (18)	60 (21)
Female	333 (58)	157 (54)	176 (62)
Ethnic group			
White	521 (91)	257 (88)	264 (93)
Ethnic minority	54 (9)	35 (12)	19 (7)
Highest educational qualification			
A Level or higher	399 (69)	208 (71)	191 (68)
GCSE, standard grade or other	152 (26)	71 (24)	81 (29)
No formal qualification	24 (4)	13 (5)	11 (4)
Marital status			
Married or living as married	233 (41)	128 (44)	105 (37)
Single	249 (43)	123 (42)	126 (45)
Separated, divorced or widowed	93 (16)	41 (14)	52 (18)
CIS-R depression diagnosis	306 (53)	162 (55)	144 (51)
CIS-R GAD diagnosis	255 (44)	133 (46)	122 (43)
CIS-R total score			
0–11	114 (20)	55 (19)	59 (21)
12–19	157 (27)	83 (28)	74 (26)
20–49	304 (53)	154 (53)	150 (53)
CIS-R depression duration			
Less than 2 years	390 (68)	197 (67)	193 (68)
2 years or more	186 (32)	95 (33)	91 (32)
Depression in the past	462 (80)	232 (79)	230 (81)
Antidepressant in the past	346 (60)	177 (61)	169 (60)
	Mean (s.d.)		
CIS-R depressive symptoms	10.43 (4.81)	10.53 (4.79)	10.33 (4.84)
GAD-7 total score	9.16 (5.20)	9.21 (5.18)	9.12 (5.22)

*Note.* CIS-R, Clinical Interview Schedule Revised; GAD, generalised anxiety disorder; GAD-7, Generalised Anxiety Disorder Assessment. Missing data for *n* = 1 in sertraline group on ethnic group, highest educational qualification, marital status, CIS-R depression diagnosis, CIS-R total score, depression in the past, and CIS-R depression score.

ECAT word categorisation was generally good at baseline (median per cent correct 93%, IQR 15%), but some participants did not understand the task or the words (21% scored below 80% correct; online Supplement). Participants made more positive than negative hits and false alarms at each time. All hits and false alarms increased from baseline to 2 weeks and then decreased at 6 weeks. Positive and negative hits and false alarms were similar across groups at 2 and 6 weeks (Table 2).

**Table 2.** Word recall (positive and negative hits and false alarms) at two and six weeks according to treatment allocation

	Placebo		Sertraline	
	*n*	Mean (s.d.)	*n*	Mean (s.d.)
Positive hits				
Baseline	291	3.03 (1.73)	284	2.88 (1.77)
2 weeks	281	3.11 (2.00)	272	2.95 (1.98)
6 weeks	276	2.31 (1.62)	260	2.32 (1.60)
Negative hits				
Baseline	291	1.93 (1.38)	284	2.07 (1.50)
2 weeks	281	2.09 (1.45)	272	2.07 (1.49)
6 weeks	275	2.04 (1.70)	259	2.00 (1.62)
Positive false alarms				
Baseline	291	1.29 (1.42)	284	1.33 (1.37)
2 weeks	281	1.60 (1.61)	272	1.55 (1.45)
6 weeks	276	1.30 (1.49)	260	1.55 (1.67)
Negative false alarms				
Baseline	291	0.87 (1.16)	284	0.91 (1.27)
2 weeks	281	0.88 (1.14)	272	0.94 (1.13)
6 weeks	276	0.94 (1.27)	260	1.10 (1.37)

*Note.* s.d., standard deviation. Hits were words that were initially presented in the categorisation task and correctly recalled. False alarms were any words falsely recalled by participants (words not presented in the categorisation task). Missing data for *n* = 1 in placebo group at baseline.

We found no evidence that treatment allocation was associated with positive hits (adjusted hits ratio = 0.98, 95% CI 0.90–1.06, *p* = 0.56; Table 3). There was no evidence that this association differed between 2 and 6 weeks (adjusted interaction hits ratio = 1.11, 95% CI 0.96–1.28, *p* = 0.18). We also found no evidence for an association with negative hits (adjusted hits ratio = 0.99, 95% CI 0.91–1.08, *p* = 0.85; Table 3), and no evidence that this association differed between 2 and 6 weeks (adjusted interaction hits ratio = 1.01, 95% CI 0.85–1.19, *p* = 0.95).

**Table 3.** Unadjusted and adjusted Poisson mixed models testing the effect of sertraline, compared with placebo, on the total number of positive or negative hits (separate models for positive and negative hits as the outcome)

	Positive hits			Negative hits		
Model	*n*	Hits ratio (95% CI)	*p* value	*n*	Hits ratio (95% CI)	*p* value
Unadjusted	576	0.98 (0.89–1.08)	0.66	576	0.99 (0.89–1.10)	0.81
Partially adjusted^a^	574	0.99 (0.91–1.07)	0.73	574	0.99 (0.91–1.08)	0.85
Fully adjusted^b^	574	0.98 (0.90–1.06)	0.56	574	0.99 (0.91–1.08)	0.85

*Note.* 95% CI, 95% confidence interval. Hits ratio can be interpreted the number of hits in the sertraline group relative to the number of hits in the placebo group.

a Adjusted for randomisation stratification variables (CIS-R total score, duration of depression, site), time, and positive and negative false alarms at all times. For positive hits as the outcome, it was also adjusted for baseline positive hits and negative hits at all times. For negative hits as the outcome, it was also adjusted for baseline negative hits and positive hits at all times.

b Partially adjusted model further adjusted for variables which were imbalanced at baseline (sex and marital status).

We then included all hits in the same Poisson mixed model, with treatment allocation and valence as exposures (Table 4). We found no evidence that treatment allocation was associated with differences in hits (adjusted hits ratio = 0.98, 95% CI 0.92–1.05, *p* = 0.61; Table 4). There was no evidence that this association differed for negative versus positive hits (adjusted interaction between treatment allocation and valence hits ratio = 1.01, 95% CI 0.90–1.13, *p* = 0.87) or across time (adjusted interaction between treatment allocation and time hits ratio = 1.07, 95% CI 0.96–1.19, *p* = 0.25). There was also no evidence that the influence of valence on the association between treatment allocation and hits differed across time-points (adjusted interaction between treatment allocation, valence and time hits ratio = 0.93, 95% CI 0.74–1.15, *p* = 0.51).

**Table 4.** Unadjusted and adjusted Poisson mixed models testing the effect of sertraline, compared with placebo, on the total number of hits

		Model 1: Effect of treatment allocation		Model 2: Interaction between treatment allocation and hits	
Models	*n*	Hits ratio (95% CI)	*p* value	Hits ratio (95% CI)	*p* value
Unadjusted	576	0.99 (0.90–1.08)	0.74	1.01 (0.91–1.13)	0.82
Partially adjusted^a^	574	0.99 (0.93–1.06)	0.75	1.01 (0.90–1.13)	0.87
Fully adjusted^b^	574	0.98 (0.92–1.05)	0.61	1.01 (0.90–1.13)	0.87

*Note.* 95% CI, 95% confidence interval. Hits ratio can be interpreted as the number of hits in the sertraline group relative to the number of hits in the placebo group.

For each model type (each row in the table), two models were run. Model 1 tested the effect of treatment allocation on hits, including valence as an exposure. Model 2 tested the interaction between treatment allocation and word valence on hits. All models included participants as a random intercept.

a Adjusted for randomisation stratification variables (CIS-R total score, duration of depression, site), time, baseline positive and negative hits, and positive and negative false alarms at all times.

b Partially adjusted model further adjusted for variables which were imbalanced at baseline (sex and marital status).

There was no evidence for a three-way interaction between treatment allocation, valence, and baseline GAD-7 score on hits (adjusted interaction hits ratio = 1.01, 95% CI 0.99–1.03, *p* = 0.47). However, there was weak evidence that there may be a three-way interaction between treatment allocation, valence, and baseline CIS-R depressive symptoms on hits (adjusted interaction hits ratio = 1.02, 95% CI 1.00–1.05, *p* = 0.06). For participants with fewer depressive symptoms, sertraline may have decreased negative hits compared to placebo. For those with more depressive symptoms, sertraline may have increased negative hits compared to placebo. In contrast, there was no evidence that there was a differential effect of sertraline on positive hits according to depressive symptom severity (Table 5).

**Table 5.** Descriptive statistics and adjusted Poisson mixed models for each stratum from the treatment allocation × valence × baseline CIS-R depressive symptom severity interaction on the hits ratio for sertraline relative to placebo group at 2 and 6 weeks

	Placebo			Sertraline			Sertraline *v*. Placebo
	Baseline	2 weeks	6 weeks	Baseline	2 weeks	6 weeks	
	Mean (s.d.) hits						Hits ratio (95% CI)
Positive hits							
Low depressive symptoms	3.12 (1.77)	3.18 (2.10)	2.45 (1.69)	3.02 (1.97)	2.87 (2.05)	2.26 (1.48)	0.92 (0.79–1.06)
Moderate depressive symptoms	2.80 (1.65)	2.78 (1.86)	2.01 (1.45)	3.00 (1.76)	3.19 (1.94)	2.43 (1.70)	1.05 (0.90–1.23)
High depressive symptoms	3.12 (1.74)	3.29 (2.00)	2.40 (1.66)	2.67 (1.57)	2.86 (1.94)	2.31 (1.63)	1.00 (0.88–1.13)
Negative hits							
Low depressive symptoms	1.86 (1.43)	1.99 (1.50)	2.15 (1.91)	2.10 (1.46)	1.86 (1.74)	1.85 (1.47)	0.92 (0.77–1.10)
Moderate depressive symptoms	1.74 (1.31)	2.09 (1.43)	1.91 (1.66)	2.19 (1.55)	2.27 (1.36)	2.13 (1.89)	0.95 (0.80–1.12)
High depressive symptoms	2.12 (1.37)	2.16 (1.42)	2.06 (1.56)	1.95 (1.50)	2.10 (1.30)	2.04 (1.51)	1.07 (0.93–1.23)

*Note.* 95% CI, 95% confidence interval. Hits ratio can be interpreted the number of hits in the sertraline group relative to the number of hits in the placebo group. All models are fully adjusted for randomisation stratification variables (CIS-R total score, duration of depression, site), time, baseline positive and negative hits, positive and negative false alarms at all times, and variables which were imbalanced at baseline (sex and marital status).

In online supplementary sensitivity analyses, there was no evidence that the number of days after randomisation participants started taking their medication or adherence to medication altered our findings. Repeating the main analysis only for patients who performed well on the ECAT at baseline also did not change the findings. Additionally, there was no evidence that changes in depressive or generalised anxiety symptoms were correlated with changes in positive or negative hits (online Supplement).

## Discussion

In a large pragmatic trial of antidepressants in primary care, we found no evidence that sertraline affected the recall of either positive or negative words, compared with placebo. We found some very weak evidence that sertraline may have reduced negative hits in people with fewer baseline depressive symptoms, but sertraline may have led to an increase in negative hits among those with more severe baseline symptoms. As this finding was not replicated with positive hits, it is unclear why sertraline had a varying effect on negative hits according to depressive symptom severity. Our findings do not provide evidence in support of the cognitive neuropsychological model of antidepressant action.

### Strengths and limitations

PANDA was the largest individual placebo-controlled trial of an antidepressant not funded by the pharmaceutical industry. The sample was pragmatic, recruited on the basis of clinical uncertainty, and depressive symptoms ranged from mild to severe. We were able to test the effect of sertraline on recall across the entire range of depressive symptom severity, in participants with and without diagnoses of depression and GAD. Our recruitment strategy should therefore have resulted in a sample that is more generalisable to the population who actually receive antidepressants within primary care.

Sertraline has a similar pharmacological profile to other SSRIs, and acts via a similar mechanism (Cipriani et al., 2018). We would therefore expect our results to apply to other SSRIs. Generalising these findings to other antidepressant classes is more difficult, although the cognitive neuropsychological model does propose that emotional processing is a common mechanism of action across antidepressants (Harmer et al., 2017).

Recall performance was relatively poor but was similar to other studies of patients with depression (Harmer et al., 2009; Lewis et al., 2017). Our task used some uncommon words (e.g. poised, neurotic) which may not have been understood by some participants. Fluency in English was not assessed but participants had to be able to understand and complete study questionnaires to be eligible. We do not think this influenced our findings, which were not altered when we restricted the sample to those who performed the ECAT well at baseline. However, the use of emotional processing tasks in samples that are more representative than select groups of healthy volunteers, where language and understanding may differ, must be considered.

Participants performed the recall task three times. Different adjectives were presented at each time, but the recall test was not a surprise at 2 and 6 weeks. Participants may have attempted to memorise words whilst completing the initial categorisation task, which could have reduced any effects of sertraline compared to placebo on recall. However, in another study using the same task over three times, associations with depressive symptoms were similar across all times and recall did not improve with time (Lewis et al., 2017). We also did not find evidence that recall improved over time in this study. Participants may not be able to consciously change biases in recall despite being aware of the test.

### Findings in context

The PANDA trial found that sertraline reduced anxiety symptoms after 6 weeks, before any effect on depressive symptoms. These unexpected results have been discussed elsewhere (Lewis et al., 2019). Although the modest effect of sertraline on depressive symptoms after 6 weeks may have affected our findings, the effect of sertraline on anxiety symptoms should also have occurred through modification of emotional processing biases (Harmer et al., 2017; Reinecke & Harmer, 2016; Roiser et al., 2012). We thus expected to find evidence for differences in recall at 6 weeks according to treatment allocation, despite the lack of effect of sertraline on depressive symptoms. However, we found no evidence that sertraline (compared to placebo) affected recall after 2 or 6 weeks. We therefore did not find evidence to support the cognitive neuropsychological model of antidepressant action.

In this study, we investigated explicit recall of positive and negative information, which is associated with depressive symptoms (Lewis et al., 2017) but may not be associated with anxiety symptoms (Reinecke & Harmer, 2016). There is evidence that biases in implicit memory for the threat are associated with anxiety symptoms (Reinecke & Harmer, 2016). Antidepressants may work by reducing automatic biases in memory for threatening information, rather than increasing the recall of positive relative to negative information. This has not been tested, but there is evidence that SSRIs modify other biases in processing threat, such as interpretation of ambiguous information as threatening (Mogg, Baldwin, Brodrick, & Bradley, 2004) and attentional vigilance to threatening facial expressions (Murphy, Yiend, Lester, Cowen, & Harmer, 2009).

The antidepressant mechanism of action may be through other forms of emotional processing, which are not involved in recall. For example, another large study used change in facial emotion recognition in an algorithm to predict antidepressant response, but adding word categorisation or recall did not improve predictions (Browning et al., 2019). Facial emotion recognition tasks may be particularly sensitive to changes in emotional processing because the ability to recognise human emotions is fundamental to social interactions (Godlewska, 2019).

Our results challenge some assumptions of the cognitive neuropsychological model of antidepressant action. It remains unclear whether, and which type of, emotional processing biases have a mechanistic role in the action of antidepressants. Antidepressants are amongst the most widely used medications in the world and understanding how antidepressants might work could lead to better indications for their use and help in the development of new treatments.

## Supplementary material

For supplementary material accompanying this paper visit https://doi.org/10.1017/S0033291720004985. click here to view supplementary material
