# Supplementary material for: The effect of sertraline on emotional processing: secondary analyses of the PANDA randomised controlled trial
Source: Psychol Med. 2021 Jan 12;52(13):2814–21. doi: 10.1017/S0033291720004985 (PMC9647512; doi:10.1017/S0033291720004985)
Supplement: Supplementary file 1 [file S0033291720004985sup001.pdf]

Supplementary Materials

Methods

ECAT performance

We checked performance on the emotional categorisation task (ECAT) at baseline by analysing the percentage of 'correct' responses. In this task, participants responded whether they would 'like' or 'dislike' to hear someone describing them using each positive and negative word. Participants' ratings were classified as correct if they chose 'like' for positive words or 'dislike' for negative words. We then calculated the percent correct out of the 40 words presented in total. As percent correct was very negatively skewed, we used the median and interquartile range to describe the average. This was done to check that participants understood the words presented.

We then repeated the main analysis limiting the sample to participants who scored 80% and above on the ECAT at baseline. This approach has been used in previous studies (e.g., Harmer et al., 2011, 2013) as, if people do not initially categorise words correctly, it may indicate that they have not understood the words or completed the task properly. If participants did not initially see the words, the recall test would not be a valid test of memory. We therefore tested the association between treatment allocation and positive and negative recall in participants who performed at 80% and above on the baseline ECAT.

Delays starting medication

Due to delays between randomisation and starting medication, we tested whether the effect of sertraline on recall differed according to how long after randomisation participants started taking their medication. We tested an interaction between treatment allocation, valence, and number of days after randomisation participants started taking their medication in the fully adjusted model.

Adherence

We tested whether adherence to medication altered associations by repeating all fully adjusted analyses only for those who met adherence criteria (80% adherence) at two and six weeks.

Previous antidepressant use

We examined whether the effect of sertraline on recall differed according to whether participants had previously taken antidepressants. We tested whether there was evidence for an interaction between treatment allocation and previous antidepressant use on positive hits or negative hits (separate outcomes in two fully adjusted Poisson regression models).

Change in recall and symptoms
In exploratory analyses, we tested the relationship of change in positive and negative hits with change in depressive and anxiety symptoms in the sertraline group. Depressive symptoms were measured using the Patient Health Questionnaire (PHQ-9; Kroenke et al., 2001) and symptoms of generalised anxiety were measured with the Generalised Anxiety Disorder Assessment (GAD-7; Spitzer et al., 2006). Both were tested alongside the incidental recall task at baseline and 6 weeks after randomisation.

We calculated a change score for depressive symptoms by taking the baseline PHQ-9 score from the PHQ-9 score at 6 weeks for each participant in the sertraline group. A negative change score meant that symptoms were more severe at baseline, so decreased from baseline to 6 weeks. In contrast, a positive change score meant that symptoms increased from baseline to 6 weeks. This method was repeated for generalised anxiety symptoms, positive hits, and negative hits. We then tested Pearson product-moment correlations between each symptom change score and positive and negative hits to assess whether changes in recall were related to changes in symptoms.

Results

ECAT performance
ECAT data were available for 559 participants at baseline (n=16 (3%) missing due to technical errors). Number of correct responses ranged from 0 to 40, with the median percent correct 93% (interquartile range 83% to 98%). Only 29 participants (5%) scored less than 50% on the ECAT (accuracy less than chance). Accuracy did not differ between placebo (median = 93%, IQR=85% to 98%) and sertraline (median = 93%, IQR = 80% to 98%) groups. This indicates that participants generally understood the words presented and the recall task was a valid test of memory.

We then repeated the main analysis just for participants who performed well on the ECAT task at baseline (accuracy at 80% or above). As in the main analysis for the whole sample, there was no evidence that treatment allocation was associated with differences in positive or negative hits (Supplementary Table 1).

Delays starting medication
Delays between randomisation and starting medication meant that follow-ups were carried out a median of one week and five weeks after medication was started. Patients started medication a median of eight days after randomisation (IQR 6–11, range 1–70; reported by 548 (95%) participants). There was no evidence for an interaction between treatment allocation, valence and number of days after randomisation participants started taking their medication on hits (adjusted interaction p=0.85).

Adherence

On average, adherence was greater than 80% at all times and we observed no difference in adherence between groups (Lewis et al., 2019). Repeating all analyses only for patients who met medication adherence criteria (n=408; 71% of total sample) did not alter the findings. In the fully adjusted model, we found no evidence that treatment allocation was associated with positive hits (adjusted hits ratio=0.98, 95% CI=0.89 to 1.08, p=0.69) or negative hits (adjusted hits ratio=1.01, 95% CI=0.91 to 1.11, p=0.92).

Including all hits in the same Poisson mixed model, we found no evidence that treatment allocation was associated with differences in hits (adjusted hits ratio=0.99, 95% CI=0.92 to 1.08, p=0.87). There was also no evidence that this association differed for negative versus positive hits for patients who met medication adherence criteria (adjusted interaction between treatment allocation and valence p=0.84).

Previous antidepressant use

Overall, 60% of participants reported taking an antidepressant in the past, and this did not differ between study arms (placebo=61%, sertraline=60%). In the fully adjusted model, we found no evidence for an interaction between treatment allocation and previous antidepressant use on positive hits (adjusted interaction p=0.14) or negative hits (adjusted interaction p=0.72).

Change in recall and symptoms

On average, depressive symptoms decreased from baseline to 6 weeks in the sertraline group (depressive symptom change score M=-3.49, SD=5.55), as did generalised anxiety symptoms (change score M=-3.50, SD=5.19). Positive hits on the recall task also decreased from baseline to 6 weeks (positive hits change score M=-0.50, SD=1.97). Negative hits did not change substantially over time (negative hits change score M=-0.06, SD=1.65). There was no evidence that changes in either depressive or generalised anxiety symptoms were correlated with changes in either positive or negative hits (Supplementary Table 2).

References

Harmer, C. J., Dawson, G. R., Dourish, C. T., Favaron, E., Parsons, E., Fiore, M., Zucchetto, M., Bifone, A., Poggesi, I., Fernandes, S., Alexander, R. C., & Goodwin, G. M. (2013). Combined NK(1) antagonism and serotonin reuptake inhibition: effects on emotional processing in humans. *J Psychopharmacol*, *27*(5), 435–443. https://doi.org/10.1177/0269881112472558

Harmer, C. J., De Bodinat, C., Dawson, G. R., Dourish, C. T., Waldenmaier, L., Adams, S., Cowen, P. J., & Goodwin, G. M. (2011). Agomelatine facilitates positive versus negative affective processing in healthy volunteer models. In *Journal of Psychopharmacology* (Vol. 25, Issue 9, pp. 1159–1167). https://doi.org/10.1177/0269881110376689

Kroenke, K., Spitzer, R. L., & Williams, J. B. W. (2001). The PHQ-9: validity of a brief depression severity measure. *J Gen Intern Med*, *16*(9), 606–613.

Lewis, G., Duffy, L., Ades, A. E., Amos, R., Araya, R., Brabyn, S., Button, K. S., Churchill, R., Derrick, C., Dowrick, C., Gilbody, S., Fawsitt, C., Hollingworth, W., Jones, V., Kendrick, T., Kessler, D., Kounali, D. Z., Khan, N., Lanham, P., … Lewis, G. (2019). The clinical effectiveness of sertraline in primary care and the role of depression severity and duration (PANDA): a pragmatic , double-blind , placebo-controlled randomised trial. *The Lancet Psychiatry*. https://doi.org/10.1016/S2215-0366(19)30366-9

Spitzer, R. L., Kroenke, K., Williams, J. B. W., & Löwe, B. (2006). A brief measure for assessing generalized anxiety disorder: The GAD-7. *Archives of Internal Medicine*, *166*(10), 1092–1097. https://doi.org/10.1001/archinte.166.10.1092

Supplementary Table 1. *Unadjusted and adjusted Poisson mixed models testing the hits ratio in positive hits and negative hits for sertraline relative to placebo group at two and six weeks, just for participants who performed at 80% accuracy and above on the baseline ECAT.*

**Model**	**Positive hits**			**Negative hits**		
	n	Hits ratio (95% CI)	p value	n	Hits ratio (95% CI)	p value
**Unadjusted**	439	0.94 (0.85 to 1.04)	0.26	439	0.98 (0.87 to 1.09)	0.67
**Partially adjusted**[a]	439	0.96 (0.88 to 1.04)	0.31	439	0.98 (0.89 to 1.08)	0.71
**Fully adjusted**[b]	439	0.95 (0.87 to 1.04)	0.25	439	0.98 (0.89 to 1.08)	0.73

Note. 95% CI: 95% confidence interval. Hits ratio can be interpreted the number of hits in the sertraline group relative to the number of hits in the placebo group.

[a] Adjusted for randomisation stratification variables (CIS-R total score, duration of depression, site), time, and positive and negative false alarms at all times. For positive hits as the outcome, it was also adjusted for baseline positive hits and negative hits at all times. For negative hits as the outcome, it was also adjusted for baseline negative hits and positive hits at all times.

[b] Partially adjusted model further adjusted for variables which were imbalanced at baseline (sex and marital status).

Supplementary Table 2. *Pearson product-moment correlations between change in depressive and generalised anxiety symptoms from baseline to 6 weeks and change in positive and negative hits from baseline to 6 weeks in the sertraline group.*

	**Change in depressive symptoms**			**Change in generalised anxiety symptoms**		
	n	Correlation coefficient	p value	n	Correlation coefficient	p value
**Change in positive hits**	267	0.006	0.93	264	-0.06	0.33
**Change in negative hits**	267	-0.07	0.24	264	-0.07	0.28
